# Patient-Reported Outcomes from a Randomized, Active-Controlled, Open-Label, Phase 3 Trial of Burosumab Versus Conventional Therapy in Children with X-Linked Hypophosphatemia

**DOI:** 10.1007/s00223-020-00797-x

**Published:** 2021-01-23

**Authors:** Raja Padidela, Michael P. Whyte, Francis H. Glorieux, Craig F. Munns, Leanne M. Ward, Ola Nilsson, Anthony A. Portale, Jill H. Simmons, Noriyuki Namba, Hae Il Cheong, Pisit Pitukcheewanont, Etienne Sochett, Wolfgang Högler, Koji Muroya, Hiroyuki Tanaka, Gary S. Gottesman, Andrew Biggin, Farzana Perwad, Angela Williams, Annabel Nixon, Wei Sun, Angel Chen, Alison Skrinar, Erik A. Imel

**Affiliations:** 1 Department of Paediatric Endocrinology, Royal Manchester Children’s Hospital, Manchester, UK; 2 Faculty of Biology, Medicine and Health, University of Manchester, Manchester, UK; 3 Shriners Hospitals for Children —Washington University School of Medicine in St Louis, St Louis, MO USA; 4 Shriners Hospital for Children — Canada, McGill University, Montreal, QC Canada; 5 The University of Sydney Children’s Hospital Westmead Clinical School, The Children’s Hospital at Westmead, Westmead, NSW Australia; 6 Department of Endocrinology, The Children’s Hospital at Westmead, Westmead, NSW Australia; 7 Department of Pediatrics, University of Ottawa, Ottawa, ON Canada; 8 Division of Endocrinology and Metabolism, Children’s Hospital of Eastern Ontario, Ottawa, ON Canada; 9 Division of Pediatric Endocrinology & Center for Molecular Medicine, Karolinska Institute, Stockholm, Sweden; 10 School of Medical Sciences, Örebro University, Örebro, Sweden; 11 Department of Pediatrics, University of California, San Francisco, San Francisco, CA USA; 12 Departments of Pediatrics, Division of Endocrinology and Diabetes, Vanderbilt University School of Medicine, Vanderbilt University, Nashville, TN USA; 13 Department of Pediatrics, Osaka Hospital, Japan Community Healthcare Organization, Osaka, Japan; 14 Department of Pediatrics, Osaka University Graduate School of Medicine, Osaka, Japan; 15 Seoul National University Children’s Hospital, Seoul, Republic of Korea; 16 Center of Endocrinology, Diabetes and Metabolism, Children’s Hospital Los Angeles, Los Angeles, CA USA; 17 Department of Paediatrics, Hospital for Sick Children, Toronto, ON Canada; 18 Department of Paediatrics and Adolescent Medicine, Johannes Kepler University Linz, Linz, Austria; 19 Institute of Metabolism and Systems Research, University of Birmingham, Birmingham, UK; 20 Department of Endocrinology and Metabolism, Kanagawa Children’s Medical Center, Yokohama, Japan; 21 Okayama Saiseikai General Hospital Outpatient Center, Okayama, Japan; 22 Shriners Hospitals for Children, St Louis, MO USA; 23 Kyowa Kirin International, Marlow, UK; 24 Chilli Consultancy, Salisbury, UK; 25 Kyowa Kirin Pharmaceutical Development, Princeton, NJ USA; 26 Ultragenyx Pharmaceutical, Novato, CA USA; 27 Department of Medicine and Department of Pediatrics, Indiana University School of Medicine, Indianapolis, IN USA

**Keywords:** Burosumab, X-linked hypophosphatemia, Patient-reported outcomes, Patient-reported outcomes measurement information system

## Abstract

Changing to burosumab, a monoclonal antibody targeting fibroblast growth factor 23, significantly improved phosphorus homeostasis, rickets, lower-extremity deformities, mobility, and growth versus continuing oral phosphate and active vitamin D (conventional therapy) in a randomized, open-label, phase 3 trial involving children aged 1–12 years with X-linked hypophosphatemia. Patients were randomized (1:1) to subcutaneous burosumab or to continue conventional therapy. We present patient-reported outcomes (PROs) from this trial for children aged ≥ 5 years at screening (*n* = 35), using a Patient-Reported Outcomes Measurement Information System (PROMIS) questionnaire and SF-10 Health Survey for Children. PROMIS pain interference, physical function mobility, and fatigue scores improved from baseline with burosumab at weeks 40 and 64, but changed little with continued conventional therapy. Pain interference scores differed significantly between groups at week 40 (− 5.02, 95% CI − 9.29 to − 0.75; *p* = 0.0212) but not at week 64. Between-group differences were not significant at either week for physical function mobility or fatigue. Reductions in PROMIS pain interference and fatigue scores from baseline were clinically meaningful with burosumab at weeks 40 and 64 but not with conventional therapy. SF-10 physical health scores (PHS-10) improved significantly with burosumab at week 40 (least-squares mean [standard error] + 5.98 [1.79]; *p* = 0.0008) and week 64 (+ 5.93 [1.88]; *p* = 0.0016) but not with conventional therapy (between-treatment differences were nonsignificant). In conclusion, changing to burosumab improved PRO measures, with statistically significant differences in PROMIS pain interference at week 40 versus continuing with conventional therapy and in PHS-10 at weeks 40 and 64 versus baseline.

**Trial registration**: ClinicalTrials.gov NCT02915705

**Supplementary information:**

The online version of this article (10.1007/s00223-020-00797-x) contains supplementary material, which is available to authorized users.

## Introduction

X-linked hypophosphatemia (XLH) is a rare, heritable, lifelong phosphate-wasting disease. Loss-of-function mutations in the *PHEX* (phosphate-regulating endopeptidase homologue, X-linked) gene result in characteristic elevation of circulating fibroblast growth factor 23 (FGF23) levels, leading to reduced renal phosphate reabsorption and decreased production of active vitamin D (1,25[OH]_2_D) manifesting as chronic hypophosphatemia and impaired mineralization of bones and teeth, as well as muscle weakness [1, 2].

XLH typically manifests in early childhood as rickets, skeletal deformities, short stature, and in some children with dental abscesses [1–4]. Untreated or inadequately treated children commonly suffer impaired mobility and physical function, such as delayed walking, unusual gait, muscle weakness, bone, joint, and muscle pain, and emotional and social challenges [2–4]. Short stature acquired in childhood and skeletal deformities are irreversible, without surgery, after completion of growth. Elevated circulating FGF23 and hypophosphatemia persist into adulthood; adults often develop pseudofractures, fractures, enthesopathies, hyperparathyroidism, and early-onset osteoarthritis, and suffer increasing pain, stiffness, and loss of physical function [3].

For the past 40 years, therapy for XLH has primarily consisted of multiple daily doses of oral phosphate supplementation with active vitamin D (calcitriol or alfacalcidol; “conventional therapy”). Oral phosphate preparations can be unpalatable and can cause gastrointestinal symptoms, and the frequent dosing regimen is burdensome for patients and their caregivers [2, 3, 5]. Furthermore, regular monitoring and appropriate dose adjustments are needed to minimize the risk of complications of conventional therapy including nephrocalcinosis and hyperparathyroidism [1–3, 5].

Burosumab is a fully human monoclonal antibody (IgG1) that binds to FGF23 and inhibits its activity [6, 7]. The efficacy of burosumab in pediatric XLH has been demonstrated by increasing the levels of serum phosphorus in two phase 2 studies (UX023-CL201, NCT02163577; UX023-CL205, NCT02750618), as well as in children with rickets (rickets severity score [RSS] ≥ 2.0) in the randomized phase 3 trial of burosumab versus continued conventional therapy (UX023-CL301, NCT02915705). These trials demonstrated improvement in clinical outcomes, including rickets, lower-limb deformities, and mobility, as assessed by the 6-min walk test, with the CL301 trial demonstrating superiority of changing to burosumab over continuation of conventional therapy [2, 8, 9].

In addition to clinical outcomes, patient-reported outcome (PRO) data were collected in the phase 3 pediatric trial using Patient-Reported Outcomes Measurement Information System (PROMIS) instruments, the Short Form-10 (SF-10) Health Survey for Children, and the Faces Pain Scale—Revised (FPS-R) to quantify the impairment and compare their evolution on burosumab versus continued conventional therapy. Pain is prevalent in pediatric patients with XLH and is most frequently observed in the lower limbs, but also occurs in the back and hips [4]. Children with XLH often have trouble performing age-appropriate gross motor activities, such as walking, running, and jumping, due to bowing of the femur, tibia, and fibula, and tibial rotation that causes the feet to turn in towards each other. Gait disturbance is common in pediatric patients with XLH, reported in > 80% of children [4].

Here, we present the PRO results from the phase 3 pediatric trial, specifically the endpoints of PROMIS pain interference, physical function mobility, and fatigue; health-related quality of life (HRQoL) according to SF-10; and pain intensity according to FPS-R. We also conducted a subgroup analysis to investigate whether PRO scores varied with rickets severity, sex, geographic region, or *PHEX* disease-causing variant.

## Subjects and Methods

This open-label, randomized, active-controlled, phase 3 trial was conducted at 16 international sites with experience treating XLH. The institutional review board at each participating center approved the protocol. The trial was conducted in accordance with the Declaration of Helsinki and the Good Clinical Practice guidelines developed at the International Conference on Harmonization of Technical Requirements for Registration of Pharmaceuticals for Human Use. Trial design details have been published previously [9] and are only briefly described here.

Eligibility criteria for this trial were as follows: age 1–12 years when informed consent was obtained; confirmed diagnosis of XLH according to fasting serum phosphorus < 0.97 mmol/L (3.0 mg/dL); a confirmed *PHEX* disease-causing variant or a variant of unknown significance in the patient, or in a directly related family member with appropriate X-linked dominant inheritance; radiographic evidence of rickets in the wrist and/or knee; a total Thacher RSS ≥ 2.0; and prior treatment with conventional therapy for ≥ 6 consecutive months (children aged < 3 years) or ≥ 12 consecutive months (children aged ≥ 3 years) up until enrollment. Parents or guardians provided written informed consent for their children to participate, and children gave written assent according to local guidelines. Patients were randomly assigned (1:1) to receive subcutaneous burosumab (starting dose 0.8 mg/kg every 2 weeks) or continue conventional therapy with oral phosphate and active vitamin D, the doses of which were titrated and individualized based on published recommendations [1, 6]. Patients received study medication for up to 64 weeks. PRO instruments were completed at baseline and at weeks 24, 40, and 64.

### Patient-Reported Outcome Assessments

PROs were assessed using linguistically validated, approved instruments during the trial for patients aged ≥ 5 years only at the screening visit. PROMIS is a set of measures developed by the US National Institutes of Health to evaluate physical, mental, and social health [10, 11]. A fixed-length short-form PROMIS instrument comprising the pain interference, physical function mobility, and fatigue domains was created by selecting items from the three pediatric item banks of PROMIS (version 2.0; Online Resource, Table 2). Pain interference consisted of four items scored using five response options indicating the frequency of pain interference (“never,” “almost never,” “sometimes,” “often,” and “almost always”); physical function mobility consisted of 10 items scored using five response options to indicate severity (“with no trouble,” “with a little trouble,” “with some trouble,” “with a lot of trouble,” and “not able to do”); and fatigue consisted of eight items scored using five response options indicating the severity (“never,” “almost never,” “sometimes,” “often,” and “almost always”).

Items were selected using information from qualitative interviews of five children with XLH aged 8–12 years and their parents: one parent of an 8-year-old child with XLH and four parents of children with XLH aged 5–7 years. Item appropriateness was based on evidence from the concept elicitation, conceptual mapping, and cognitive debriefing exercises conducted as part of the qualitative interviews. The pain interference, physical function mobility, and fatigue items were well understood and found to be relevant and appropriate assessments of those concepts for the children and their parents. These qualitative data therefore established content validity of the selected PROMIS domains. An analysis of the psychometric properties of the PROMIS instrument (both the self-reported form for children aged ≥ 8 years and the parent proxy report form for children aged 5 to < 8 years) consisting of the three domains in the current trial (*n* = 35; children aged 5–12 years) confirmed it to be a reliable, valid, and responsive method for use in clinical trials in children with XLH [12].

The PROMIS domains were each measured at study assessment visits using a recall period of the previous 7 days. Children aged ≥ 8 years at screening completed a self-reported PROMIS instrument. For children aged 5 to < 8 years at screening, the parent or legal guardian completed the parent proxy version of the PROMIS instrument throughout the trial. PROMIS data were uploaded to the PROMIS online scoring system [13] to obtain the final scores for each domain. All raw scores generated from the PROMIS instrument were translated into standardized scores (termed T-scores), based on a calibration sample resulting in a calibration population mean of 50 and standard deviation (SD) of 10. The calibration sample consisted of a US cohort of 4,129 children aged 8–17 years, with 55% aged 8–12 years. The majority of children in the calibration sample (94%) were recruited from hospital-based general pediatric and subspecialty clinics, with 6% from school settings; 35% had consulted a clinician for a chronic illness diagnosis or treatment within 6 months, and 9% had two or more chronic illnesses (the most common chronic conditions were asthma, affecting 18% of children, followed by attention-deficit disorder/attention-deficit hyperactivity disorder [ADHD], arthritis, and gastrointestinal disorders) [14]. Higher scores on the PROMIS pain interference and fatigue domains indicate greater detriment (i.e., worse pain and more fatigue), whereas a higher score on the PROMIS physical function mobility domain indicates less detriment (i.e., better physical function mobility).

Overall HRQoL was assessed using the SF-10 Health Survey for Children, a validated 10-item, caregiver-completed questionnaire designed to assess physical and psychosocial HRQoL in healthy and ill children. Each question has five response options (“Excellent,” “Very good,” “Good,” “Fair,” and “Poor”) with a recall period of the past 4 weeks. Responses were used to generate two component summary scores: physical health score (PHS-10) and psychosocial health score (PSS-10), scored according to published methods [15]. The scale was scored so that a score of 50 corresponds to the average score in a 2006 sample, which comprised a combination of children from the general population and a supplemental sample with disability and chronic conditions; higher global scores are associated with better HRQoL.

The FPS-R was used to assess current pain at each study visit. The self-reported scale uses graphical facial representations of pain to allow self-reporting of current pain intensity at rest (not during or immediately after physical activity) on a 0–10 scale (0 = no hurt to 10 = hurts worst; even numbers only) and has been validated in children aged 5–16 years [16].

### Statistical Analysis

The primary endpoint for this trial, namely change in rickets severity from baseline to week 40, has been reported previously [9]. Changes from baseline in PROMIS scores were assessed as secondary endpoints, and changes from baseline in SF-10 PHS-10 and PSS-10 were assessed as exploratory endpoints. Here, we report data for changes to week 64 to provide longer-term information on XLH in children. SAS® software version 9.4 or higher (SAS Institute, Cary, NC, USA) was used for statistical analyses.

PRO endpoints were analyzed using a generalized estimating equation repeated-measures analysis. Treatment, visit, and interaction of treatment-by-visit were included as categorical variables, and baseline measures and baseline total RSS stratification factor were included as independent variables in the model. Total RSS was used in this model, as it has been validated in 52 children with XLH, with higher total RSS associated with greater impairment in walking ability as assessed by the 6-min walk test [17]. Data for model-based estimates of the changes from baseline, standard error (SE), and corresponding 95% confidence intervals (CIs) were assessed for significance at the 5% level. Missing data were treated as missing for all analyses. Only data for patients with a baseline measurement and at least one post-baseline measurement were included in analyses of change from baseline.

As the trial was not powered to assess differences in between-group changes beyond the primary endpoint, within-group changes from baseline were reported for variables with nonsignificant between-group differences. A change of 2–3 points is considered clinically meaningful for PROMIS pediatric scales [18]. To interpret within-group trial results, clinically relevant differences were explored based on a minimally important difference (MID) threshold of 3 points [19].

In an exploratory analysis, testing for treatment-by-subgroup was used to detect treatment effect heterogeneity across four subgroups (RSS: ≤ 2.5, > 2.5; sex: male, female; region: Japan, rest of world; *PHEX* status: clearly pathogenic *PHEX* variants, or likely pathogenic/variant of unknown significance) on the PRO domains using week 40 and week 64 data.

## Results

PRO assessments were completed (by the child or their parent/guardian) for all participants aged ≥ 5 years at screening, which included 15/29 patients in the burosumab group and 20/32 patients in the conventional therapy group. Baseline assessments occurred at randomization for all participants after a 7-day washout period from prior conventional therapy. Baseline characteristics for all participants (patients aged 1–12 years at screening) and those aged ≥ 5 years at screening are presented in Table 1. Overall, baseline characteristics were similar between the two age cohorts; however, serum 1,25(OH)_2_D concentration was lower in the cohort aged ≥ 5 years than in the total patient group (mean ± SD: 91 ± 36 vs 103 ± 43 pmol/L, respectively), particularly in the burosumab group (96 ± 38 vs 110 ± 48 pmol/L) (Table 1). Patients aged ≥ 5 years had received conventional therapy for a mean of 5.54 years.

**Table 1:** Patient demographic and baseline characteristics for participants ≥ 5 years at screening and for all patients (aged 1–12 years)

Characteristic	Patients aged ≥ 5 years at screening			All patients		
	Burosumab (*n* = 15)	Conventional therapy (*n* = 20)	Total (*n* = 35)	Burosumab (*n* = 29)	Conventional therapy (*n* = 32)	Total (*n* = 61)
Age, years,						
*Mean (SD)*	8.6 (2.4)	8.4 (2.2)	8.5 (2.2)	5.8 (3.4)	6.3 (3.2)	6.3 (3.3)
Sex, *n* (%)						
*Boys*	10 (66.7)	9 (45.0)	54.3)	13 (44.8)	14 (43.8)	27 (44.3)
*Girls*	5 (33.3)	11 (55.0)	16 (45.7)	16 (55.2)	18 (56.3)	34 (55.7)
Ethnic origin, *n* (%)						
*White*	12 (80.0)	14 (70.0)	26 (74.3)	25 (86.2)	25 (78.1)	50 (82.0)
*Asian*	1 (6.7)	5 (25.0)	6 (17.1)	2 (6.9)	6 (18.8)	8 (13.1)
*Other*	1 (13.3)	1 (5.0)	3 (8.6)	2 (6.9)	1 (3.1)	3 (4.9)
Geographic region, *n* (%)						
*Japan*	1 (6.7)	3 (15.0)	4 (11.4)	2 (6.9)	3 (9.4)	5 (8.2)
*ROW*	14(93.3)	17(85.0)	31 (88.6)	27 (93.1)	28 (90.6)	56 (91.8)
TmP/GFR, mmol/L						
*Mean (SD)*	0.67 (0.12)	0.66 (0.11)	0.67 (0.11)	0.65 (0.11)	0.71 (0.12)	0.68 (0.12)
Height Z score						
*Mean (SD)*	− 2.4 (1.2)	− 1.9 (0.8)	− 2.1 (1.0)	− 2.3 (1.2)	− 2.1 (0.9)	− 2.2 (1.0)
*Median (min, max)*	− 2.0 (− 5.0, − 0.5)	− 2.1 (− 3.1, − 0.1)	− 2.0 (− 5.0, − 0.1)	− 2.3 (− 3.1, − 1.5)	− 2.1 (− 2.5, − 1.4)	− 2.2 (− 5.0, − 0.1)
Weight, Z score						
*Mean (SD)*	− 1.0 (1.4)	− 0.4 (0.8)	− 0.9 (1.2)	− 0.9 (1.2)	− 0.6 (0.9)	− 0.8 (1.0)
*Median (min, max)*	− 0.8 (− 3.2, 1.3)	− 0.6 (− 1.6, 1.5)	− 0.8 (− 3.2, 1.4)	− 0.8 (− 3.2, 1.4)	− 0.7 (− 2.3, 1.5)	− 0 8 (− 3.2, 1.5)
Serum phosphorus concentration, mmol/L						
*Mean (SD)*	0.75 (0.07)	0.73 (0.09)	0.74 (0.08)	0.78 (0.08)	0.74 (0.08)	0.76 (0.08)
Serum 1,25(OH)2D concentration, pmol/L						
*Mean (SD)*	96 (38)	89 (36)	91 (36)	110 (48)	96 (36)	103 (43)
Alkaline phosphatase concentration, U/L						
*Mean (SD)*	493.3 (146.4)	516.8 (168.1)	506.7 (157.3)	510.8 (124.9)	523.4 (154.4)	517.4 (140.2)
Total Thacher RSS						
*Mean (SD)*	3.1 (0.8)	3.0 (0.9)	3.0 (0.8)	3.2 (1.0)	3.2 (1.1)	3.2 (1.1)
*Median (min, max)*	3.0 (2.0, 5.0)	3.0 (2.0, 4.5)	3.0 (2.0, 5.0)	3.0 (2.5, 4.0)	3.0 (2.5, 3.5)	3.0 (2.0, 6.5)

*Max* maximum, *min* minimum, *ROW* rest of world, *RSS* Rickets Severity Score, *SD* standard deviation, *TmP/GFR* tubular maximum for phosphate reabsorption per glomerular filtration rate

### PROMIS Pain Interference

**Fig. 1:**
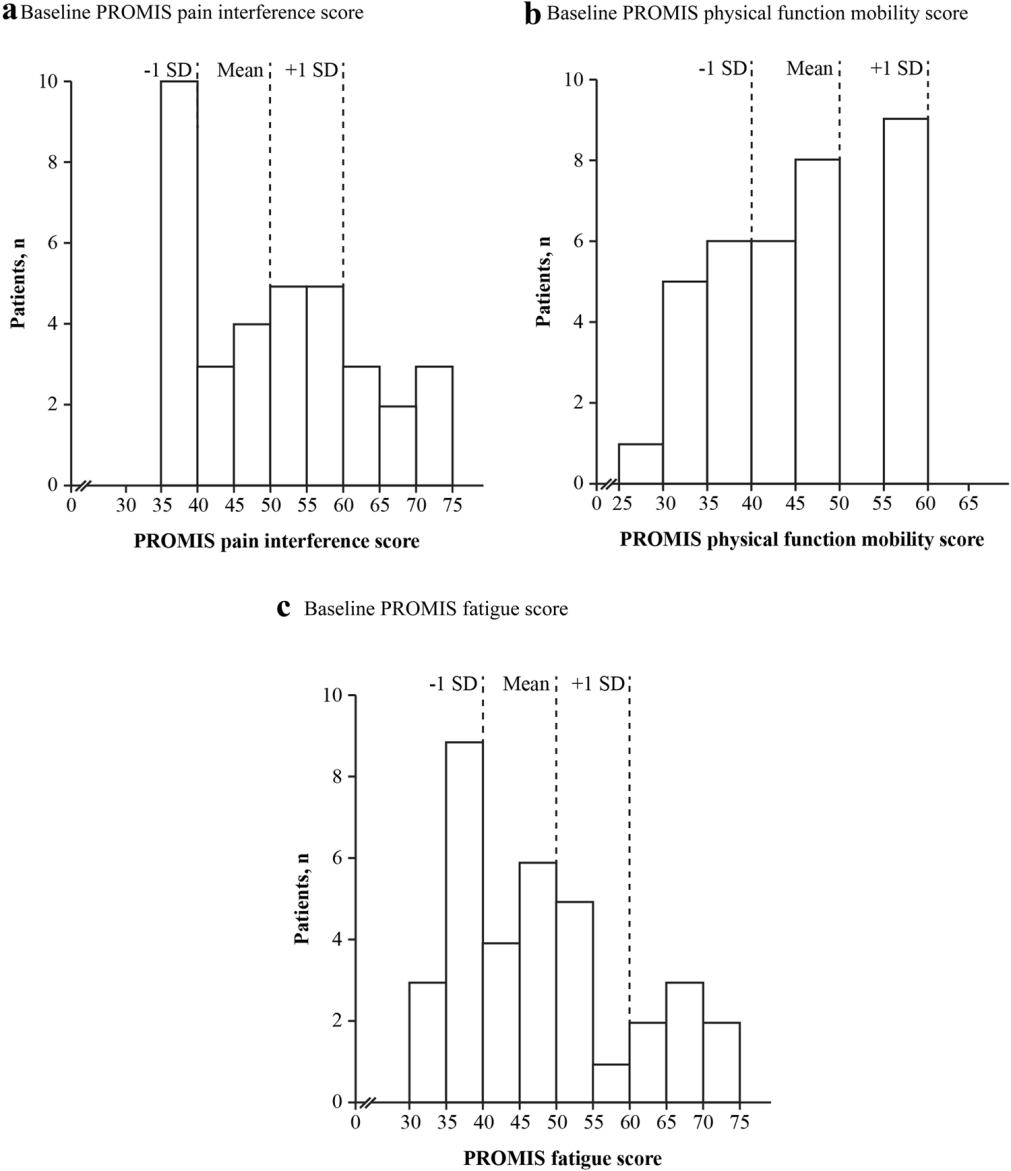
Baseline PROMIS (**a**) pain interference, (**b**) physical function mobility, and (**c**) fatigue scores for patients aged ≥ 5 years (*n* = 35). Data show standardized PROMIS T-scores; higher T-scores indicate more pain interference, better function mobility, and worse fatigue. Reference lines at ± 1 SD of the mean are based on a population mean of 50 and SD of 10. *PROMIS* Patient-Reported Outcomes Measurement Information System, *SD* standard deviation

**Fig. 2:**
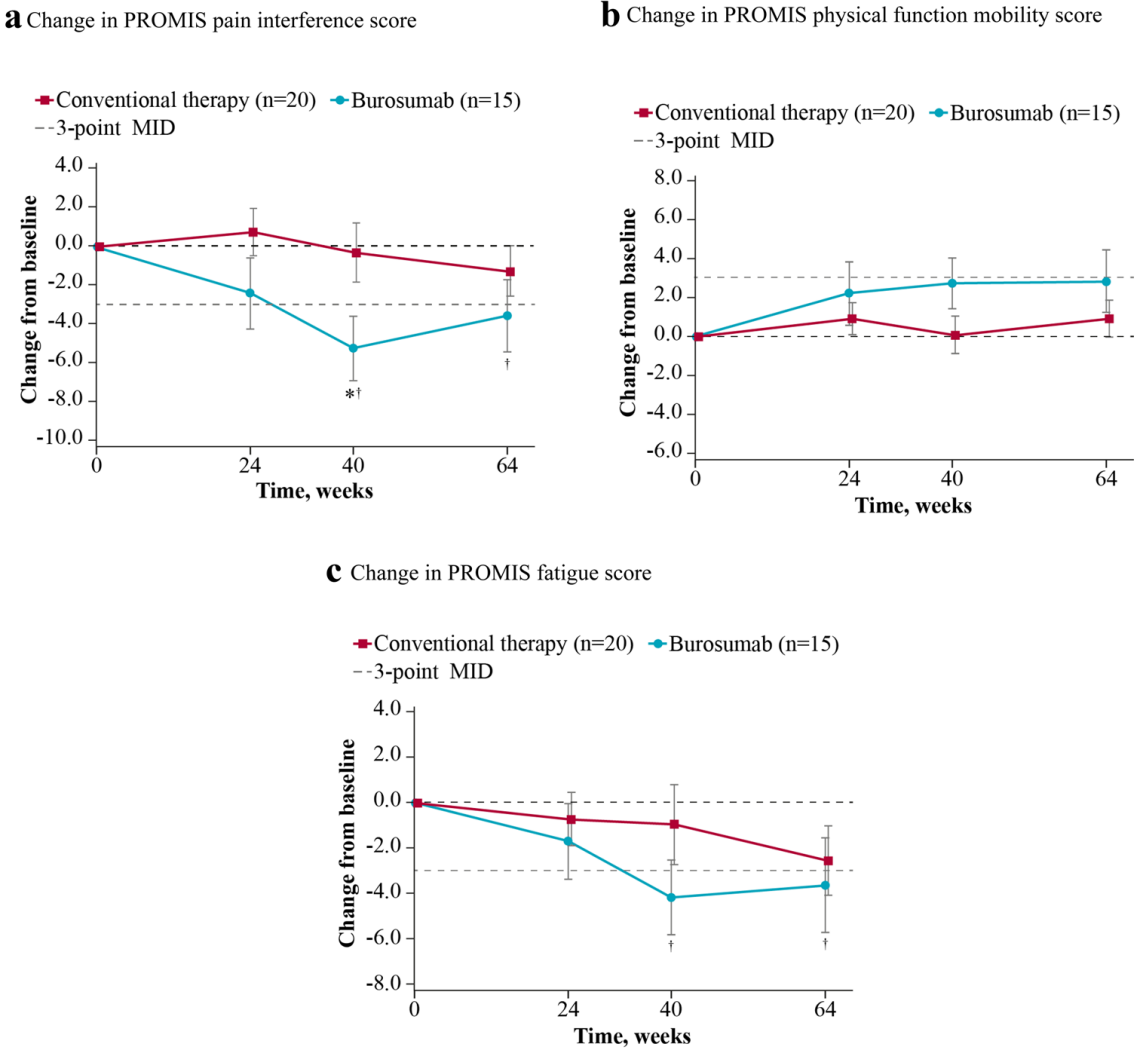
Change from baseline in PROMIS (**a**) pain interference, (**b**) physical function mobility, and (**c**) fatigue scores for patients aged ≥ 5 years (*n* = 35). Data are expressed as LS mean (standard error). *p < 0.05 for LS mean change at week 40 (burosumab–conventional therapy). ^†^Indicates the mean change is ≥ 3-point MID from baseline. *LS* least-squares, *MID* minimally important difference, *PROMIS* Patient-Reported Outcomes Measurement Information System

Higher scores on the pain interference domain reflect pain having a greater impact on daily activities, with decreases in scores reflecting improvements in this domain. At baseline, the mean ± SD pain interference T-score was 53.1 ± 10.95 for burosumab and 49.9 ± 12.02 for continued conventional therapy, broadly comparable to the mean of the calibration sample of 50 [14]. Eight of the 35 patients (23%) had pain interference scores ≥ 1 SD higher (worse) than the calibration sample average (Fig. 1a). Pain interference score decreased from baseline in the burosumab group (least-squares [LS] mean [SE] change: − 5.31 [1.705] at week 40 and − 3.55 [1.873] at week 64), indicating reduced levels of pain interference, but changed little in the group who continued to receive conventional therapy (− 0.29 [1.539] at week 40 and − 1.29 [1.267] at week 64). The change in pain interference score exceeded the 3-point MID threshold in patients receiving burosumab at both weeks 40 and 64, consistent with a clinically meaningful reduction in pain interference for this group. The change from baseline in the group continuing conventional therapy was less than the 3-point MID threshold. Burosumab was associated with a significantly greater change from baseline than conventional therapy at week 40 (between-group difference − 5.02, 95% CI − 9.29 to − 0.75; *p* = *0*.0212) but not at week 64 (− 2.26, 95% CI − 6.61 to + 2.09; *p* = *0*.3091) (Fig. 2a). Descriptive item-level results at baseline and week 64 can be found in the appendix (Online Resource, Table 3).

### PROMIS Physical Function Mobility

Higher scores on the PROMIS physical function mobility domain indicate less detriment (i.e., better physical function mobility), with increases in scores reflecting improvements in this domain. At baseline, the mean ± SD physical function mobility T-score was 45.2 ± 9.05 for burosumab and 45.5 ± 9.86 for continued conventional therapy, similar to the mean of 50 of the calibration sample [14]. Twelve of the 35 patients (34%) had impaired physical function indicated by physical function mobility scores ≥ 1 SD lower (worse) than the calibration sample average (Fig. 1b). Physical function mobility score increased numerically from baseline in the burosumab group (LS mean [SE]: + 2.78 [1.336] at week 40 and + 2.82 [1.648] at week 64), indicating improved physical function mobility, but showed little change in the continued conventional therapy group (+ 0.10 [0.966] at week 40 and + 0.92 [0.962] at week 64). Neither group had achieved a meaningful change from baseline based on a 3-point MID. Differences between treatment groups were not statistically significant at either week 40 (+ 2.68, 95% CI − 0.52 to + 5.89; *p* = *0*.1009) or week 64 (+ 1.90, 95% CI − 1.80 to + 5.59; *p* = *0*.3145) (Fig. 2b). Descriptive item-level results at baseline and week 64 can be found in the appendix (Online Resource, Table 3).

### PROMIS Fatigue

Higher scores on the PROMIS fatigue domain reflect greater levels of fatigue, with decreases in scores reflecting improvements in this domain. At baseline, the mean ± SD fatigue T-score was 48.8 ± 9.60 for burosumab and 47.0 ± 13.70 for continued conventional therapy, similar to the mean of 50 of the calibration sample [14]. Seven of the 35 patients (20%) had fatigue scores at least 1 SD higher (worse) than the calibration sample average (Fig. 1c). Fatigue scores decreased from baseline to weeks 40 and 64 in burosumab-treated patients (LS mean [SE], − 4.29 [1.709] at week 40 and − 3.65 [2.119] at week 64), reflecting reduced levels of fatigue, but showed little change in those continuing to receive conventional therapy (LS mean [SE], − 1.05 [1.754] at week 40 and − 2.57 [1.547] at week 64). Meaningful change from baseline was achieved for the burosumab group at weeks 40 and 64, based on a 3-point MID. There was no meaningful change for the conventional therapy group at weeks 40 or 64. Between-group differences did not reach statistical significance at either week 40 (− 3.25, 95% CI − 7.86 to + 1.37; *p* = *0*.1676) or week 64 (− 1.08, 95% CI − 6.21 to + 4.06; *p* = *0*.6810) (Fig. 2c). Descriptive item-level results at baseline and week 64 can be found in the appendix (Online Resource, Table 3).

### SF-10 Health Survey for Children

**Fig. 3:**
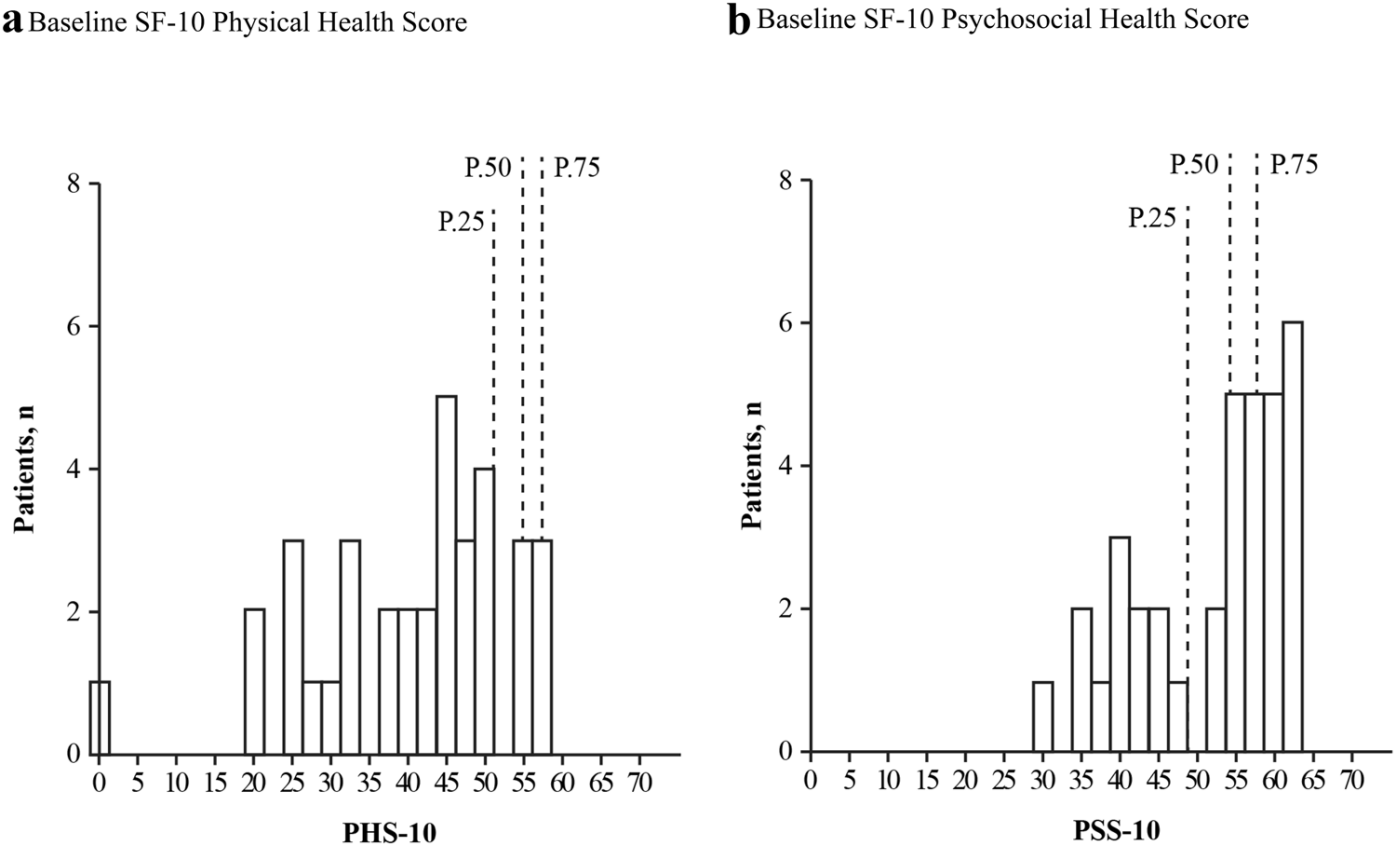
Baseline SF-10 Health Survey for Children (**a**) PHS-10 and (**b**) PSS-10 for patients aged ≥ 5 years (*n* = 35). P.25, P.50, and P.75 are the 25th, 50th, and 75th percentiles from the general population. Higher global scores indicate better HRQoL. *HRQoL* health-related quality of life, *PHS-10* physical health score, *PSS-10* psychosocial health score, *SF-10* Short Form-10

**Fig. 4:**
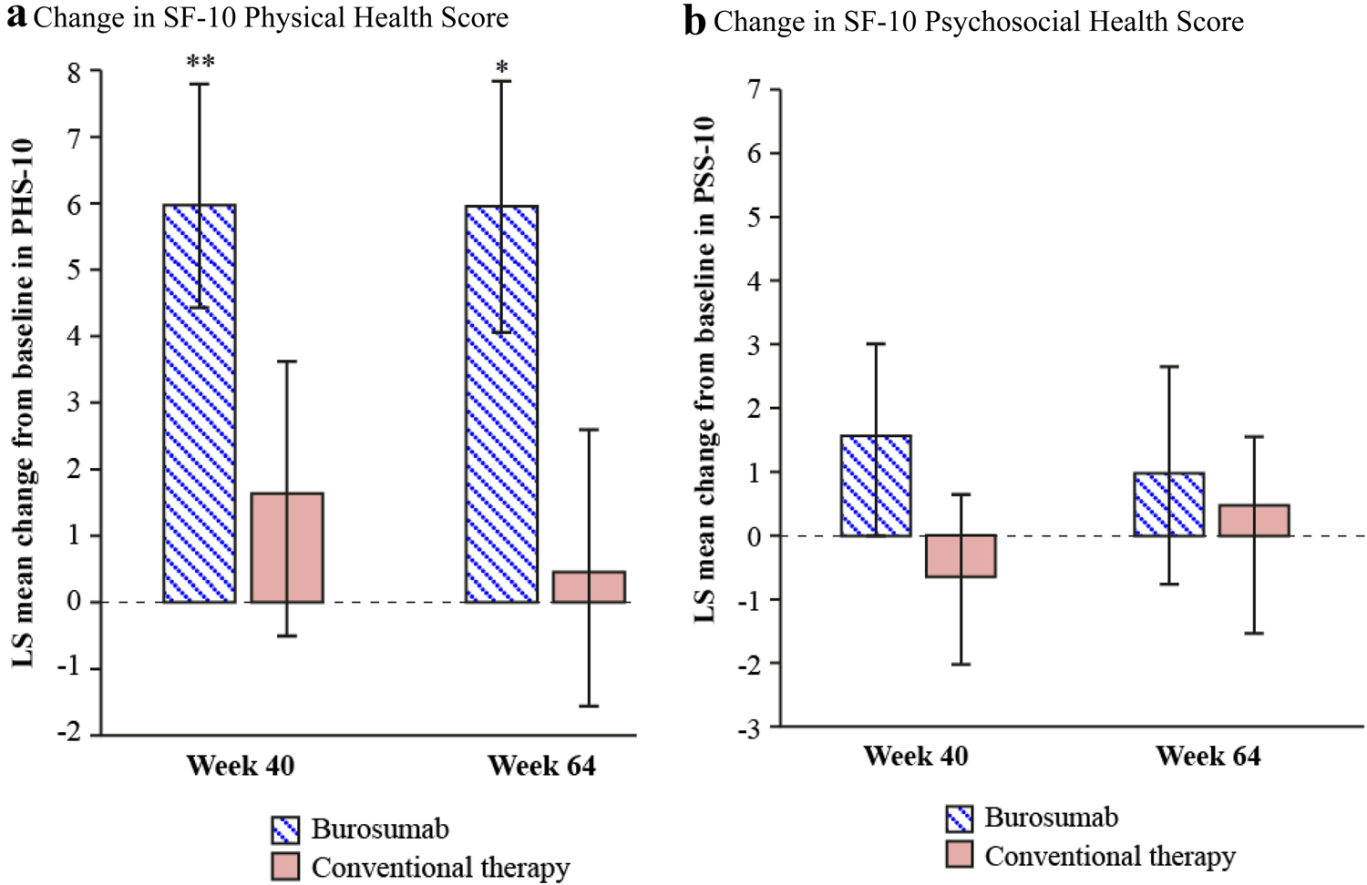
Changes from baseline to weeks 40 and 64 for SF-10 Health Survey for Children (**a**) PHS-10 and (**b**) PSS-10 for all patients aged ≥ 5 years (*n* = 35). Data are expressed as LS mean ± standard error. *p < 0.01 for change from baseline to week 64; **p < 0.001 for change from baseline to week 40. *LS* least-squares, *PHS-10* physical health score, *PSS-10* psychosocial health score, *SF-10* Short Form-10

The trial population had baseline scores below the 25th percentile for PHS-10 (mean [SD] score; 40.47 [13.14] total population, 40.03 [10.07] burosumab, 40.74 [15.30] conventional therapy) and below the 50th percentile for PSS-10 (mean [SD] score; 51.92 [9.42] total population, 50.76 [9.65] burosumab, 52.79 [9.40] conventional therapy) (Fig. 3). Patients receiving burosumab showed significant improvements in LS mean (SE) PHS-10 scores from baseline at both week 40 (+ 5.98 [1.79]; *p* = *0*.0008) and week 64 (+ 5.93 [1.88]; *p* = *0*.0016) (Fig. 4a). By contrast, there were no statistically significant changes from baseline in the conventional therapy group at week 40 (+ 1.65 [2.17]) or week 64 (+ 0.44 [2.22]). The LS mean (SE) differences between the burosumab and continued conventional therapy groups did not meet statistical significance at either week 40 (+ 4.33 [2.82]) or week 64 (+ 5.49 [2.91]). There were no statistically significant changes in LS mean (SE) PSS-10 scores from baseline to weeks 40 or 64 for either burosumab (+ 1.53 [1.52] and + 0.94 [1.176], respectively) or continued conventional therapy (− 0.66 [1.38] and + 1.44 [1.21], respectively), with no significant differences between the two treatment groups (LS mean difference [SE]: + 2.19 [2.10] at week 40; − 0.50 [2.12] at week 64) (Fig. 4b).

### Faces Pain Scale—Revised

Most children in both treatment groups reported no pain at baseline, week 40, or week 64 according to the FPS-R. Median FPS-R scores were 0 at all time points for both groups, and there were no significant differences between groups (*p* = *0*.8786).

### Subgroup Analyses

Treatment effect heterogeneity was assessed for the trial population in predefined subgroups: RSS: ≤ 2.5 (*n* = 22), > 2.5 (*n* = 39); sex: male (*n* = 27), female (*n* = 34). Data by region (Japan [*n* = 5], rest of world [*n* = 56]) and *PHEX* status (clearly pathogenic *PHEX* variant [*n* = 55], or PHEX variants that were likely pathogenic/variant of unknown significance [*n* = 6]) were removed from this analysis because of the imbalance in patient numbers in these groups. No statistically significant interactions (*p* < 0.05) were identified among the five PRO domains for the RSS and sex subgroup categories.

## Discussion

Children with XLH whose PRO scores indicate pain interference, fatigue, physical function mobility impairment, and reduced HRQoL despite conventional therapy would benefit from a disease-modifying treatment that alleviates the disorder’s long-term physical and psychosocial complications [4]. Our phase 3 CL301 trial demonstrated that burosumab results in greater improvement in phosphorus homeostasis, growth, lower-extremity deformities, and healing of rickets in children with XLH compared with continuing conventional therapy [9]. In the present analyses, we determined the impact of switching from conventional therapy to burosumab treatment compared to continuing conventional therapy on PROs of pain interference, physical function mobility, fatigue, and HRQoL.

Baseline PROMIS pain interference, physical function mobility, and fatigue scores were similar to the PROMIS calibration sample, which included 4,129 children primarily from hospital-based general pediatric and subspecialty clinics. In this sample, 35% of children had consulted a clinician for a chronic illness diagnosis or treatment within 6 months, and 9% had two or more chronic illnesses [14]. Baseline PROMIS pain interference, physical function mobility, and fatigue scores were similar to those reported for other lifetime diseases that start in childhood, such as sickle cell disease, Crohn disease, juvenile arthritis, juvenile dermatomyositis, chronic kidney disease, or systemic lupus erythematosus [20–27].

Baseline SF-10 scores indicate impaired HRQoL in the present trial population, especially with regard to physical health, with lower baseline PHS-10 scores (mean score, 40.03) than those seen in children with asthma, attention-deficit hyperactivity disorder, depression, or learning disabilities (mean range, 43.83–48.23) [12]. However, SF-10 is a caregiver-completed questionnaire, which may underestimate the child’s health and well-being [28]. The FPS-R tool is validated to assess current pain, though it has not been psychometrically validated specifically in children with XLH. At all study visits, the XLH patients indicated no pain, suggesting that the FPS-R assessment of current pain at rest may not reliably assess the burden of pain severity in this population.

In patients who received burosumab, the decrease from baseline in pain interference was significantly greater than in those who continued conventional therapy, although only up to week 40. The improvement with burosumab was not fully sustained from week 40 to week 64 but did not return to baseline levels. The lack of a persistent benefit with burosumab up to week 64 may be explained by small patient numbers and possible increased activity-related pain for patients who had received early benefit from burosumab. Furthermore, pain and its associated impact in XLH is complex and multifactorial [1, 4], and expected improvements in bone health with burosumab may not be sufficient to fully address all aspects of pain syndrome over a short period of time.

Improvements from baseline in the PROMIS physical function mobility and fatigue domains were also achieved with burosumab versus conventional therapy, although the differences between groups were not statistically significant. Patients receiving burosumab did have clinically meaningful changes (based on 3-point MIDs) in two of the three PROMIS domains (pain interference and fatigue) by week 40, which were maintained at week 64. Burosumab also significantly improved the physical health domain (PHS-10) by approximately 10% at week 40, which was maintained at week 64. In patients receiving conventional therapy, changes in physical health from baseline were not seen; differences between treatment groups did not reach statistical significance, although the trial was not powered to show differences in these endpoints. There were no statistically significant interactions among the five PRO domains for the predefined subgroup categories investigated (RSS and sex).

Improvements in PROs are reflected by mobility data from the 6-min walk test reported in the primary manuscript [9]. These data showed that patients randomized to burosumab had significantly greater improvements from baseline in percent predicted distance walked over 6 min than those continuing to receive conventional therapy at week 64 (LS mean change from baseline 9% vs 2%; 95% CI 0.01–14.52; *p* = *0*.0496) [9].

Improvements in pain interference, physical function mobility, fatigue, and HRQoL may be explained by the mechanism of action of burosumab. Burosumab addresses the deficiency of serum phosphate by directly binding to FGF23 and inhibiting its signaling, increasing tubular phosphate reabsorption, as well as increasing serum 1,25(OH)_2_D levels and increasing gastrointestinal phosphate absorption [29]. Increased serum phosphate levels result in improved bone mineralization [29], improved muscular function [30], and restoration of ATP synthesis [31–33], and thus ultimately may manifest as improvements in patient-reported symptoms, function, and HRQoL.

In relation to PRO measurements, this trial has several limitations. Our trial was not powered to assess between-group differences for secondary and exploratory outcomes, such as PROs, nor for subgroup analyses. For example, sample size was insufficient to explore whether higher phosphate concentration or rapidity of correction of alkaline phosphatase reflected PRO measurements. Therefore, it remains unknown whether mid-range serum phosphate levels improve PROs to a greater extent than low-normal values. Children aged younger than 1 year or older than 12 years were not recruited, and children younger than 5 years did not complete PROs, precluding extrapolation of results to these age groups and limiting the sample size to 15 patients in the burosumab group and 20 patients in the conventional therapy group. Further, only those with RSS ≥ 2 were enrolled in this trial; thus, the baseline PRO scores or the degree of expected improvement with treatment for those with lower RSS scores is not known. This trial only randomized treatment for 64 weeks, while XLH is a lifelong chronic disease. Further follow-up is warranted to determine longer-term effects on HRQoL and patient-reported pain interference, physical function mobility, and fatigue. Furthermore, compared with the prior clinical standard of care, children on conventional treatment in this trial were perhaps more meticulously monitored and managed, because of the frequent visits and rigorous trial requirements. High levels of compliance and adherence to conventional treatment in clinical trial settings can result in greater improvements in outcomes with conventional therapy than that seen in routine standard of care [34–37].

In conclusion, in this phase 3 trial, changing from conventional therapy to burosumab was associated with numerical increases in PROMIS physical function mobility, clinically meaningful reductions in PROMIS pain interference and fatigue up to 64 weeks, and a statistically significant reduction in PROMIS pain interference up to 40 weeks. Statistically significant improvements in SF-10 PHS-10 were observed up to 64 weeks from baseline in children aged 5–12 years with XLH.

## Supplementary information

Below is the link to the electronic supplementary material.

Supplementary information 1 (PDF 163 kb)

## Data Availability

The corresponding author had full access to all the data in the study.

## References

[1] Carpenter TO, Imel EA, Holm IA, Jan de Beur SM, Insogna KL (2011). A clinician’s guide to X-linked hypophosphatemia. J Bone Miner Res.

[2] Whyte MP, Carpenter TO, Gottesman GS, Mao M, Skrinar A, San Martin J, Imel EA (2019). Efficacy and safety of burosumab in children aged 1–4 years with X-linked hypophosphataemia: a multicentre, open-label, phase 2 trial. Lancet Diabetes Endocrinol.

[3] Linglart A, Biosse-Duplan M, Briot K, Chaussain C, Esterle L, Guillaume-Czitrom S, Kamenicky P, Nevoux J, Prié D, Rothenbuhler A, Wicart P, Harvengt P (2014). Therapeutic management of hypophosphatemic rickets from infancy to adulthood. Endocr Connect.

[4] Skrinar A, Dvorak-Ewell M, Evins A, Macica C, Linglart A, Imel EA, Theodore-Oklota C, San Martin J (2019). The lifelong impact of X-linked hypophosphatemia: results from a burden of disease survey. J Endocr Soc.

[5] Collins M (2018). Burosumab: at long last, an effective treatment for FGF23-associated hypophosphatemia. J Bone Miner Res.

[6] Carpenter TO, Imel EA, Ruppe MD, Weber TJ, Klausner MA, Wooddell MM, Kawakami T, Ito T, Zhang Z, Humphrey J, Insogna KL, Peacock M (2014). Randomized trial of the anti-FGF23 antibody KRN23 in X-linked hypophosphatemia. J Clin Invest.

[7] Imel EA, Zhang X, Ruppe MD, Weber TJ, Klausner MA, Ito T, Vergeire M, Humphrey JS, Glorieux FH, Portale AA, Insogna K, Peacock M, Carpenter TO (2015). Prolonged correction of serum phosphorus in adults with X-linked hypophosphatemia using monthly doses of KRN23. J Clin Endocrinol Metab.

[8] Carpenter TO, Whyte MP, Imel EA, Boot AM, Högler W, Linglart A, Padidela R, Van't Hoff W, Mao M, Chen C-Y, Skrinar A, Kakkis E, San Martin J, Portale AA (2018). Burosumab therapy in children with X-linked hypophosphatemia. N Engl J Med.

[9] Imel EA, Glorieux FH, Whyte MP, Munns CF, Ward LM, Nilsson O, Simmons JH, Padidela R, Namba N, Cheong HI, Pitukcheewanont P, Sochett E, Högler W, Muroya K, Tanaka H, Gottesman GS, Biggin A, Perwad F, Mao M, Chen C-Y, Skrinar A, San Martin J, Portale AA (2019). Burosumab versus conventional therapy in children with X-linked hypophosphataemia: a randomised, active-controlled, open-label, phase 3 trial. Lancet.

[10] Broderick JE, DeWitt EM, Rothrock N, Crane PK, Forrest CB (2013). Advances in patient-reported outcomes: the NIH PROMIS® measures. EGEMS (Wash DC).

[11] HealthMeasures (2020) Measure Development & Research. Northwestern University. http://www.healthmeasures.net/explore-measurement-systems/promis/measure-development-research. Accessed 15 Sept 2020.

[12] Nixon A, Williams A, Skrinar A, Theodore-Oklota C (2019) Psychometric validation of the PROMIS^®^ physical function mobility, pain interference and fatigue in a cohort of paediatric X-linked hypophosphatemia (XLH) patients. Proceedings of the International Society for Pharmacoeconomics and Outcomes Research Europe, Nov 2–6, Copenhagen, Denmark.

[13] HealthMeasures (2020) PROMIS. Northwestern University. http://www.healthmeasures.net/explore-measurement-systems/promis. Accessed 15 Sept 2020.

[14] Irwin DE, Stucky BD, Thissen D, DeWitt EM, Lai JS, Yeatts K, Varni JW, DeWalt DA (2010). Sampling plan and patient characteristics of the PROMIS pediatrics large-scale survey. Qual Life Res.

[15] Saris-Baglama RN, DeRosa MA, Raczek AE, Bjorner JB, Turner-Bowker DM, Ware JE (2007). The SF-10^TM^ health survey for children: a user’s guide.

[16] Hicks CL, von Baeyer CL, Spafford PA, van Korlaar I, Goodenough B (2001). The faces pain scale-revised: toward a common metric in pediatric pain measurement. Pain.

[17] Thacher TD, Pettifor JM, Tebben PJ, Creo AL, Skrinar A, Mao M, Chen C-Y, Chang T, San Martin J, Carpenter TO (2019). Rickets severity predicts clinical outcomes in children with X-linked hypophosphatemia: utility of the radiographic rickets severity score. Bone.

[18] Thissen D, Liu Y, Magnus B, Quinn H, Gipson DS, Dampier C, Huang I-C, Hinds PS, Selewski DT, Reeve BB, Gross HE, DeWalt DA (2016). Estimating minimally important difference (MID) in PROMIS pediatric measures using the scale-judgment method. Qual Life Res.

[19] Wyrwich KW, Norquist JM, Lenderking WR, Acaster S (2013). Methods for interpreting change over time in patient-reported outcome measures. Qual Life Res.

[20] DeWalt DA, Gross HE, Gipson DS, Selewski DT, DeWitt EM, Dampier CD, Hinds PS, Huang I-C, Thissen D, Varni JW (2015). PROMIS^®^ pediatric self-report scales distinguish subgroups of children within and across six common pediatric chronic health conditions. Qual Life Res.

[21] Arvanitis M, DeWalt DA, Martin CF, Long MD, Chen W, Jaeger B, Sandler RS, Kappelman MD (2016). Patient-reported outcomes measurement information system in children with Crohn's disease. J Pediatr.

[22] Brandon TG, Becker BD, Bevans KB, Weiss PF (2017). Patient-reported outcomes measurement information system tools for collecting patient-reported outcomes in children with juvenile arthritis. Arthritis Care Res (Hoboken).

[23] Cunningham NR, Kashikar-Zuck S, Mara C, Goldschneider KR, Revicki DA, Dampier C, Sherry DD, Crosby L, Carle A, Cook KF, Morgan EM (2017). Development and validation of the self-reported PROMIS pediatric pain behavior item bank and short form scale. Pain.

[24] Dampier C, Barry V, Gross HE, Lui Y, Thornburg CD, DeWalt DA, Reeve BB (2016). Initial evaluation of the pediatric PROMIS^®^ health domains in children and adolescents with sickle cell disease. Pediatr Blood Cancer.

[25] Dampier C, Jaeger B, Gross HE, Barry V, Edwards L, Lui Y, DeWalt DA, Reeve BB (2016). Responsiveness of PROMIS^®^ pediatric measures to hospitalizations for sickle pain and subsequent recovery. Pediatr Blood Cancer.

[26] Reeve BB, Edwards LJ, Jaeger BC, Hinds PS, Dampier C, Gipson DS, Selewski DT, Troost JP, Thissen D, Barry V, Gross HE, DeWalt DA (2018). Assessing responsiveness over time of the PROMIS^®^ pediatric symptom and function measures in cancer, nephrotic syndrome, and sickle cell disease. Qual Life Res.

[27] Jones JT, Carle AC, Wootton J, Liberio B, Lee J, Schanberg LE, Ying J, DeWitt EM, Brunner HI (2017). Validation of patient-reported outcomes measurement information system short forms for use in childhood-onset systemic lupus erythematosus. Arthritis Care Res (Hoboken).

[28] Theunissen NC, Vogels TG, Koopman HM, Verrips GH, Zwinderman KA, Verloove-Vanhorick SP, Wit JM (1998). The proxy problem: child report versus parent report in health-related quality of life research. Qual Life Res.

[29] Lyseng-Williamson KA (2018). Burosumab in X-linked hypophosphatemia: a profile of its use in the USA. Drugs Ther Perspect.

[30] Veilleux LN, Cheung M, Ben Amor M, Rauch F (2012). Abnormalities in muscle density and muscle function in hypophosphatemic rickets. J Clin Endocrinol Metab.

[31] Pesta DH, Tsirigotis DN, Befroy DE, Caballero D, Jurczak MJ, Rahimi Y, Cline GW, Dufour S, Birkenfeld AL, Rothman DL, Carpenter TO, Insogna K, Petersen KF, Bergwitz C, Shulman GI (2016). Hypophosphatemia promotes lower rates of muscle ATP synthesis. FASEB J.

[32] Chen YY, Kao TW, Chou CW, Wu CJ, Yang HF, Lai CH, Wu LW, Chen WL (2018). Exploring the link between serum phosphate levels and low muscle strength, dynapenia, and sarcopenia. Sci Rep.

[33] Allen DG, Trajanovska S (2012). The multiple roles of phosphate in muscle fatigue. Front Physiol.

[34] van Onzenoort HA, Menger FE, Neef C, Verbeck WJ, Kroon AA, de Leeuw PW, van der Kuyet PHM (2011). Participation in a clinical trial enhances adherence and persistence to treatment: a retrospective cohort study. Hypertension.

[35] Carls GS, Tuttle E, Tan RD, Huyuh J, Yee J, Edelman SV, Polonsky WH (2017). Understanding the gap between efficacy in randomized controlled trials and effectiveness in real-world use of GLP-1 RA and DPP-4 therapies in patients with type 2 diabetes. Diabetes Care.

[36] Mauro MJ, Davis C, Zyczynski T, Khoury HJ (2015). The role of observational studies in optimizing the clinical management of chronic myeloid leukemia. Ther Adv Hematol.

[37] Tobert JA, Newman CB (2016). Statin tolerability: in defence of placebo-controlled trials. Eur J Prev Cardiol.

