# Supplementary material for: Patient-Reported Outcomes from a Randomized, Active-Controlled, Open-Label, Phase 3 Trial of Burosumab Versus Conventional Therapy in Children with X-Linked Hypophosphatemia
Source: Calcif Tissue Int. 2021 Jan 23;108(5):622–33. doi: 10.1007/s00223-020-00797-x (PMC8064984; doi:10.1007/s00223-020-00797-x)
Supplement: Supplementary file 1 — Supplementary information 1 (PDF 163 kb) [file 223_2020_797_MOESM1_ESM.pdf]

Title page

Title

Patient-Reported Outcomes from a Randomized, Active-Controlled, Open-Label, Phase 3 Trial of Burosumab Versus Conventional Therapy in Children with X-Linked Hypophosphatemia

Journal name

Calcified Tissue International

Authors

Raja Padidela MD, Michael P. Whyte MD, Francis H. Glorieux MD PhD, Craig F. Munns FRACP, Leanne M. Ward MD, Ola Nilsson MD, Anthony A. Portale MD, Jill H. Simmons MD, Noriyuki Namba MD Hae Il Cheong MD, Pisit Pitukcheewanont MD, Etienne Sochett MD, Wolfgang Högler MD, Koji Muroya MD, Hiroyuki Tanaka MD, Gary S. Gottesman MD, Andrew Biggin FRACP, Farzana Perwad MD, Angela Williams PhD, Annabel Nixon PhD, Wei Sun PhD, Angel Chen MS, Alison Skrinar PhD, and Erik A. Imel MD

Address correspondence to:

Dr Raja Padidela

Consultant Paediatric Endocrinologist and Metabolic Bone Diseases

Department of Paediatric Endocrinology

Manchester University NHS Foundation Trust Hospital

Oxford Road, Manchester M13 9WL

UK

Tel: 0161 7011628

Fax: 0161 7011631

E-mail: Raja.Padidela@mft.nhs.uk

Electronic Supplementary Material

Table 2 Questions from the validated fixed-length short-form PROMIS pediatric self-report (≥8 years) and parent proxy report (5 to <8 years) used in this trial

**PROMIS pediatric self-report questions**	**PROMIS parent report questions**
**Physical function mobility**. In both questionnaires, response options were "with no trouble," "with a little trouble," "with some trouble," "with a lot of trouble," and "not able to do."	
**In the past 7 days…**	**In the past 7 days…**
I could do sports and exercise that other kids my age could do.	My child could do sports and exercise that other kids his/her age could do.
I could get up from the floor.	My child could get up from the floor.
I could keep up when I played with other kids.	My child could keep up when he/she played with other kids.
I could walk up stairs without holding on to anything.	My child could walk up stairs without holding on to anything.
I have been physically able to do the activities I enjoy most.	My child has been physically able to do the activities he/she enjoys most.
I could carry my books in my backpack.	My child could carry his/her books in a backpack.
I could get down on my knees without holding on to something.	My child could get down on his/her knees without holding on to something.
I could get in and out of a car.	My child could get in and out of a car.
I could ride a bike.	My child could ride a bike.
I could walk more than one block.	My child could walk more than one block.
**Fatigue.** In both questionnaires, response options were "never," "almost never," "sometimes," "often," and "almost always."	
**In the past 7 days…**	**In the past 7 days…**
Being tired made it hard for me to play or go out with my friends as much as I'd like.	Being tired made it hard for my child to play or go out with friends as much as he/she would like.

I got tired easily.	My child got tired easily.
I was too tired to do sports or exercise.	My child was too tired to do sports or exercise.
I was too tired to do things outside.	My child was too tired to do things outside.
I was too tired to enjoy the things I like to do.	My child was too tired to enjoy the things he/she likes to do.
I felt too tired to spend time with my friends.	My child felt too tired to spend time with his/her friends.
I needed to sleep during the day.	My child needed to sleep during the day.
I was too tired to go up and down a lot of stairs.	My child was too tired to go up and down a lot of stairs.
**Pain interference.** In both questionnaires, response options were “never,” “almost never,” “sometimes,” “often,” and “almost always.”	
**In the past 7 days…**	**In the past 7 days…**
I had trouble sleeping when I had pain.	My child had trouble sleeping when he/she had pain.
It was hard for me to run when I had pain.	It was hard for my child to run when he/she had pain.
It was hard for me to walk one block when I had pain.	It was hard for my child to walk one block when he/she had pain.
It was hard to stay standing when I had pain.	It was hard for my child to stay standing when he/she had pain.

PROMIS Patient-Reported Outcomes Measurement Information System

Table 3 Item response variability in PROMIS pain interference, physical function mobility, and fatigue domains for patients receiving burosumab or conventional therapy

**PROMIS domain**	**Burosumab**					**Conventional therapy**				
	***n* (%)**	***n* (%)**	***n* (%)**	***n* (%)**	***n* (%)**	***n* (%)**	***n* (%)**	***n* (%)**	***n* (%)**	***n* (%)**
**Pain interference**	**Never**	**Almost never**	**Sometimes**	**Often**	**Almost always**	**Never**	**Almost never**	**Sometimes**	**Often**	**Almost always**
**Had trouble sleeping when I/they had pain**										
Baseline	9 (60.0)	1 (6.7)	3 (20.0)	0	2 (13.3)	10 (50.0)	3 (15.0)	5 (25.0)	1 (5.0)	1 (5.0)
Week 64	10 (66.7)	3 (20.0)	2 (13.3)	0	0	12 (60.0)	3 (15.0)	3 (15.0)	2 (10.0)	0
**Hard to run when I/they had pain**										
Baseline	4 (26.7)	1 (6.7)	7 (46.7)	0	3 (20.0)	8 (40.0)	2 (10.0)	3 (15.0)	3 (15.0)	4 (20.0)
Week 64	5 (33.3)	6 (40.0)	3 (20.0)	1 (6.7)	0	7 (35.0)	4 (20.0)	6 (30.0)	3 (15.0)	0
**Hard to walk when I/they had pain**										
Baseline	4 (26.7)	2 (13.3)	6 (40.0)	1 (6.7)	2 (13.3)	10 (50.0)	2 (10.0)	3 (15.0)	2 (10.0)	3 (15.0)
Week 64	4 (26.7)	7 (46.7)	2 (13.3)	1 (6.7)	1 (6.7)	8 (40.0)	3 (15.0)	4 (20.0)	3 (15.0)	2 (10.0)

**Hard to stand when I/they had pain**										
Baseline	8 (53.3)	1 (6.7)	3 (20.0)	1 (6.7)	2 (13.3)	11 (55.0)	4 (20.0)	2 (10.0)	1 (5.0)	2 (10.0)
Week 64	5 (33.3)	6 (40.0)	3 (20.0)	0	1 (6.7)	10 (50.0)	4 (20.0)	3 (15.0)	3 (15.0)	0
**Physical function mobility**	**Not able to do**	**With a lot of trouble**	**With some trouble**	**With a little trouble**	**With no trouble**	**Not able to do**	**With a lot of trouble**	**With some trouble**	**With a little trouble**	**With no trouble**
**I/they could do sports and exercise**										
Baseline	0	1 (6.7)	6 (40.0)	3 (20.0)	5 (33.3)	1 (5.0)	2 (10.0)	0	9 (45.0)	8 (40.0)
Week 64	0	1 (6.7)	1 (6.7)	6 (40.0)	7 (46.7)	0	1 (5.0)	4 (20.0)	5 (25.0)	10 (50.0)
**I/they could get up from the floor**										
Baseline	0	0	0	1 (6.7)	14 (93.3)	0	0	0	4 (20.0)	16 (80.0)
Week 64	0	0	1 (6.7)	0	14 (93.3)	0	0	0	5 (25.0)	15 (75.0)
**I/they could keep up when I/they played**										
Baseline	0	2 (13.3)	4 (26.7)	5 (33.3)	4 (26.7)	0	3 (15.0)	6 (30.0)	4 (20.0)	7 (35.0)
Week 64	0	0	4 (26.7)	4 (26.7)	7 (46.7)	0	1 (5.0)	3 (15.0)	7 (35.0)	9 (45.0)

**I/they could walk up stairs without holding**										
Baseline	1 (6.7)	0	2 (13.3)	4 (26.7)	8 (53.3)	1 (5.0)	2 (10.0)	2 (10.0)	2 (10.0)	13 (65.0)
Week 64	0	0	3 (20.0)	4 (26.7)	8 (53.3)	0	1 (5.0)	2 (10.0)	4 (20.0)	13 (65.0)
**I/they was/were able to do the activities I/they enjoy most**										
Baseline	0	0	1 (6.7)	8 (53.3)	6 (40.0)	1 (5.0)	0	3 (15.0)	4 (20.0)	12 (60.0)
Week 64	0	0	1 (6.7)	2 (13.3)	12 (80.0)	0	0	3 (15.0)	4 (20.0)	13 (65.0)
**I/they could carry my/their books in my/their backpack**										
Baseline	0	1 (6.7)	0	5 (33.3)	9 (60.0)	1 (5.0)	1 (5.0)	2 (10.0)	1 (5.0)	15 (75.0)
Week 64	0	0	1 (6.7)	1 (6.7)	13 (86.7)	0	0	3 (15.0)	5 (25.0)	12 (60.0)
**I/they could get down on my/their knees without holding**										
Baseline	0	1 (6.7)	0	1 (6.7)	13 (86.7)	1 (5.0)	0	1 (5.0)	4 (20.0)	14 (70.0)
Week 64	0	0	1 (6.7)	2 (13.3)	12 (80.0)	1 (5.0)	0	1 (5.0)	4 (20.0)	14 (70.0)
**I/they could get in and out of a car**										
Baseline	0	0	0	2 (13.3)	13 (86.7)	0	0	1 (5.0)	3 (15.0)	16 (80.0)
Week 64	0	0	2 (13.3)	0	13 (86.7)	0	0	0	4 (20.0)	16 (80.0)

**I/they could ride a bike**										
Baseline	0	0	1 (6.7)	1 (6.7)	13 (86.7)	1 (5.0)	1 (5.0)	3 (15.0)	3 (15.0)	12 (60.0)
Week 64	0	0	1 (6.7)	2 (13.3)	12 (80.0)	2 (10.0)	0	3 (15.0)	3 (15.0)	12 (60.0)
**I/they could walk more than one block**										
Baseline	0	1 (6.7)	1 (6.7)	4 (26.7)	9 (60.0)	3 (15.0)	2 (10.0)	0	3 (15.0)	12 (60.0)
Week 64	0	1 (6.7)	1 (6.7)	3 (20.0)	10 (66.7)	2 (10.0)	3 (15.0)	1 (5.0)	5 (25.0)	9 (45.0)
**Fatigue**	**Never**	**Almost never**	**Sometimes**	**Often**	**Almost always**	**Never**	**Almost never**	**Sometimes**	**Often**	**Almost always**
**Being tired made it hard for me/them to play**										
Baseline	8 (53.3)	4 (26.7)	1 (6.7)	1 (6.7)	1 (6.7)	10 (50.0)	3 (15.0)	5 (25.0)	2 (10.0)	0
Week 64	7 (46.7)	4 (26.7)	4 (26.7)	0	0	11 (55.0)	6 (30.0)	3 (15.0)	0	0
**I/they got tired easily**										
Baseline	4 (26.7)	5 (33.3)	4 (26.7)	2 (13.3)	0	10 (50.0)	2 (10.0)	4 (20.0)	1 (5.0)	3 (15.0)
Week 64	7 (46.7)	3 (20.0)	4 (26.7)	1 (6.7)	0	9 (45.0)	4 (20.0)	6 (30.0)	1 (5.0)	0

**I/they was/were too tired to do sports or exercise**										
Baseline	6 (40.0)	4 (26.7)	2 (13.3)	2 (13.3)	1 (6.7)	11 (55.0)	4 (20.0)	1 (5.0)	2 (10.0)	2 (10.0)
Week 64	8 (53.3)	3 (20.0)	3 (20.0)	1 (6.7)	0	11 (55)	4 (20.0)	5 (25.0)	0	0
**I/they was/were too tired to do things outside**										
Baseline	10 (66.7)	2 (13.3)	3 (20.0)	0	0	11 (55)	4 (20.0)	2 (10.0)	3 (15.0)	0
Week 64	10 (66.7)	2 (13.3)	2 (13.3)	1 (6.7)	0	13 (65.0)	2 (10.0)	5 (25.0)	0	0
**I/they was/were too tired to enjoy things I/they like**										
Baseline	9 (60.0)	4 (26.7)	2 (13.3)	0	0	14 (70.0)	2 (10.0)	1 (5.0)	3 (15.0)	0
Week 64	10 (66.7)	4 (26.7)	1 (6.7)	0	0	15 (75.0)	1 (5.0)	4 (20.0)	0	0
**I/they felt too tired to spend time with friends**										
Baseline	10 (66.7)	4 (26.7)	1 (6.7)	0	0	13 (65.0)	5 (25.0)	1 (5.0)	1 (5.0)	0
Week 64	10 (66.7)	4 (26.7)	1 (6.7)	0	0	13 (65.0)	4 (20.0)	3 (15.0)	0	0
**I/they needed to sleep during the day**										
Baseline	6 (40.0)	6 (40.0)	3 (20.0)	0	0	14 (70.0)	3 (15.0)	3 (15.0)	0	0
Week 64	9 (60.0)	2 (13.3)	4 (26.7)	0	0	13 (65.0)	4 (20.0)	3 (15.0)	0	0

**I/they was/were too tired to go up and down stairs**										
Baseline	6 (40.0)	4 (26.7)	5 (33.3)	0	0	9 (45.0)	5 (25.0)	3 (15.0)	1 (5.0)	2 (10.0)
Week 64	10 (66.7)	2 (13.3)	3 (20.0)	0	0	11 (55.0)	4 (20.0)	4 (20.0)	0	1 (5.0)

PROMIS Patient-Reported Outcomes Measurement Information System
